# Imaging of Ultraweak Spontaneous Photon Emission from Human Body Displaying Diurnal Rhythm

**DOI:** 10.1371/journal.pone.0006256

**Published:** 2009-07-16

**Authors:** Masaki Kobayashi, Daisuke Kikuchi, Hitoshi Okamura

**Affiliations:** 1 Department of Electronics and Intelligent Systems, Tohoku Institute of Technology, Sendai, Japan; 2 Department of Systems Biology, Kyoto University Graduate School of Pharmaceutical Sciences, Kyoto, Japan; 3 Department of Brain Science, Kobe University Graduate School of Medicine, Kobe, Japan; City of Hope Medical Center, United States of America

## Abstract

The human body literally glimmers. The intensity of the light emitted by the body is 1000 times lower than the sensitivity of our naked eyes. Ultraweak photon emission is known as the energy released as light through the changes in energy metabolism. We successfully imaged the diurnal change of this ultraweak photon emission with an improved highly sensitive imaging system using cryogenic charge-coupled device (CCD) camera. We found that the human body directly and rhythmically emits light. The diurnal changes in photon emission might be linked to changes in energy metabolism.

## Introduction

Bioluminescence, which is weak but visible, is sometimes produced in living organisms, such as fireflies or jellyfish, as the result of specialized enzymatic reactions that require adenosine triphosphate. However, virtually all living organisms emit extremely weak light, spontaneously without external photoexcitation [1]. This biophoton emission is categorized in different phenomena of light emission from bioluminescence, and is believed to be a by-product of biochemical reactions in which excited molecules are produced from bioenergetic processes that involves active oxygen species [1], [2]. Human body is glimmering with light of intensity weaker than 1/1000 times the sensitivity of naked eyes [3], [4]. By using a sensitive charge-coupled-device (CCD) camera with the ability to detect light at the level of a single photon, we succeeded in imaging the spontaneous photon emission from human bodies [3].

Previously, for obtaining an image, it took more than 1 hour of acquisition, which is practically impossible for the analysis of physiologically relevant biophoton emission. By improving the CCD camera and lens system, here we have succeeded in obtaining clear images using a short exposure time, comparable with the analysis of physiological phenomena. Since metabolic rates are known to change in a circadian fashion [5], [6], we investigated the temporal variations of biophoton emission across the day from healthy human body.

## Results and Discussion

**Figure 1:**
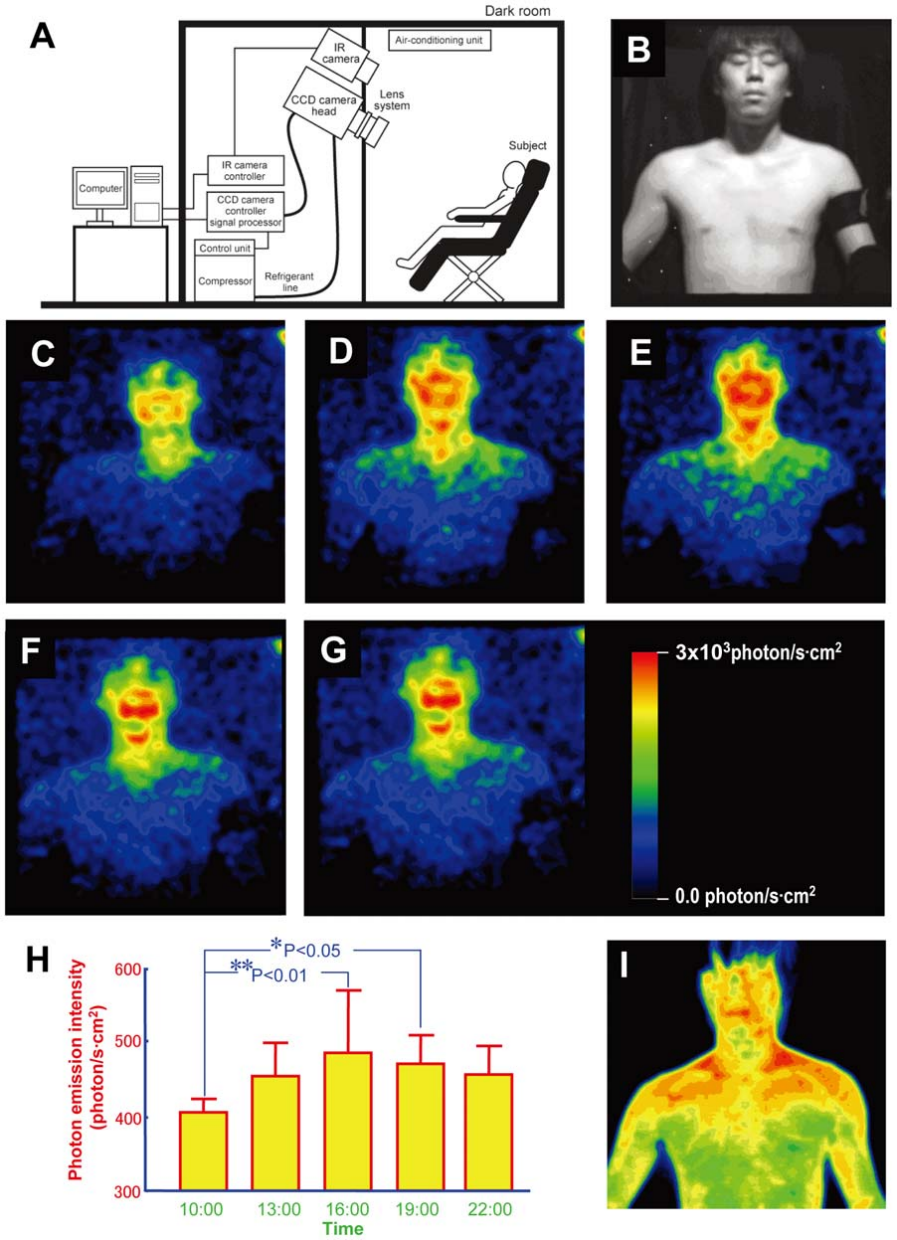
A. Schematic illustration of experimental setup. B–F. Images of ultraweak photon emission from human body. B. Image of the subject under light illumination. C. Image at 10:10. D. Image at 13:10. E. Image at 16:10. F. Image at 19:10. G. Image at 22:10 with a calibration bar which indicates the estimated radiation intensity expressed by photon number per unit of time per unit of skin surface. H. Daily rhythm of photon emission from face and body from 5 volunteers. Significant difference from the photon emission at 10:00 AM (n = 15, Mean±SD; **P<0.01, *P<0.05). I. A typical thermographic image of the subject from Fig. 1B–G.

A cooled CCD camera operated at −120°C with slow scanning mode read-out was used with a specially designed high-throughput lens system. The camera was placed in a light-tight room in complete darkness (schematic illustration of the experimental setup is shown in Fig. 1A). Five healthy male volunteers, in their 20′s, were subjected to normal light-dark conditions and allowed to sleep from 0:00–7:00. On the days of photon imaging, volunteers were kept in a room (400 lux) adjacent to the dark room. For imaging purposes, the body surface was wiped and the subject was left 15 minutes in the dark room for dark adaptation, after which the naked subject in sitting position was exposed for 20 minutes to the CCD camera. Measurements were carried out in every 3 hours from 10:00 to 22:00 and continued for 3 days. Just before and after the measurements, the surface body (thermography) and oral temperature were taken. Saliva was also collected after the photon measurements for the analysis of cortisol level as a biomarker of endogenous circadian rhythms. Temporal variation of photon emission intensity was calculated from image data with extraction of the face and body intensity.

The daily variation of photon emission is shown in Fig. 1B–G. In all images, photon emission intensity from the face was higher than from the body. Moreover, photon emission intensity from the face was not homogeneous: the central area around the mouth and the cheeks was higher than the lateral area and the orbits. Furthermore, the photon emission intensity on the face and upper body appeared to display time-dependent changes. We plotted total photon emission intensity over the body and face against time, averaged across the 5 volunteers (Fig. 1H). Photon emission was weak in the morning, increased in the afternoon and peaked in the late afternoon (ca 16:00) (one way ANOVA, F_4,74_ = 4.10, P<0.005). These data strongly suggest that there is a diurnal rhythm of photon emission from the human body. To further support this conclusion, immediately following the end of the previous experiment three volunteers were kept awake in a light (400 lux) environment and photon emission was measured at 1:00, 4:00 and 7:00 AM (Supplementary Figure S1). Photon emission formed a peak at late afternoon, then gradually decreased and stayed low at 1:00–7:00 AM in a constantly exposed light condition (400 lux), indicating the diurnal rhythm of photon might be caused by endogenous circadian mechanism.

Ultraweak biophoton emission was completely different from thermographic images showing surface temperature (Fig.1I). High photon emission were detected from the cheeks, followed by the upper neck and the forehead, while high temperature was detected in the supraclavicular lateral neck region, from which photon emission was low. In cheek, the highest level of emission reaches to 3000 photon/s·cm^2^ at 16:00 which is about double to the value at 10:00.

**Figure 2:**
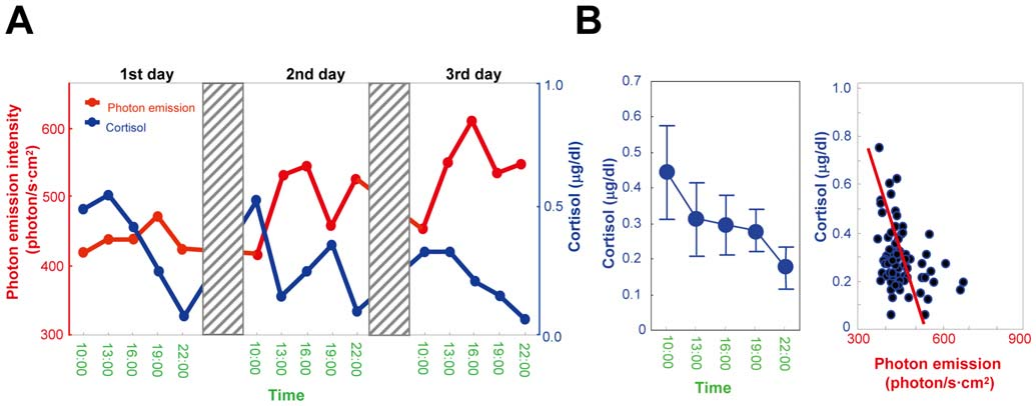
A. Comparison of temporal variation of biophoton emission intensity and cortisol concentration in saliva observed through 3 days. Shaded regions indicate sleeping time. The subject is the one of Fig. 1B–G. B. Daily change of cortisol secretion (left; n = 15, Mean±SD) and its correlation with photon emission intensity (right). A negative correlation was found (r = −0.3074, P<0.002).

Next, we examined the correlation of photon emission to other physiological parameters known to show circadian variations. In the subject of Fig.1B–G, we found a temporal decrease of cortisol from morning to evening, in opposite to the increase of photon emission (Fig. 2A). Cortisol concentration shows a clear daily rhythm, peaking in the morning and negatively correlated with photon emission intensity (p<0.002; from 5 volunteers; Fig. 2B). Body temperature, another parameter showing daily rhythms peaking at night, does not show significant correlation with photon emission (Supplementary Figure S2).

The photon emission mechanism is thought to originate from the generation of free radicals in energy metabolic processes. The spectra of photon emission detected from the palm skin span from 500 to 700 nm, with primary and secondary emission peaks at 630–670 nm and 520–580 nm, respectively [7]. Free radicals subsequently react with lipid or protein, generating electronically excited species as byproducts [1]. These excited molecules, such as carbonyl group in excited triplet state from lipid peroxidation or proteins including excited tyrosine or tryptophan, can further react with fluorophores through energy transfer and lead to photon emission [8], [9]. Higher level photon emission on facial skin might be caused by differences in the content of melanin fluorophores [10] between facial and thoracic skin.

No significant correlation of daily photon intensity and temperature was found, and the dissimilarity between photon emission and thermal image suggest that the diurnal rhythm of photon emission is not a consequence of a change of temperature or microcirculation. Moreover, a clear negative correlation of temporal changes of photon emission and cortisol might suggest that the diurnal rhythm of photon emission reflects the changes of cellular metabolic processes under the control of the circadian clock. Circadian rhythms are generated in most cells throughout the body, driven by clock genes interlocked in transcription/translation feedback loops [11], [12]. Recent advances of chronobiology have revealed that the redox state of the cells regulates circadian gene expression, indicating the importance of metabolic cues for clock oscillations [6], [13], [14]. Indeed, glucose utilization, accompanied by oxygen consumption, shows robust rhythms in the main mammalian circadian center [5]. By the regulation of cellular respiratory chain producing reactive oxygen species, which in turns react with molecules including proteins, lipids and fluorophores, whose excited states emit biophotons [1], [8], [9], [10], the human body glitters to the rhythm of the circadian clock.

## Materials and Methods

### CCD camera system

Spectral Instruments 600 series CCD camera system (Spectral Instruments, Inc., AZ, USA) was used. Mounted CCD42-40 (e2v technologies Ltd., Essex, UK), which is a back-illuminated, full-frame operation CCD with a 2048×2048 pixel resolution and 13.5×13.5 µm pixel size. The camera system is equipped with a cooling head to maintain the CCD at −120°C using a closed-cycle mechanical cryogenic unit. Under these conditions, quantum efficiency is 75% at the peak wavelength. Dark current is 0.65 electron/pixel/h and readout noise in the slow scanning mode is less than 4.5 electron rms. The CCD camera head has a specially designed lens system, which is designed to maximize the light collection efficiency (numerical aperture (NA) of the lens system on the detector side is 0.5 and the number of lenses is restricted 7 pieces). Magnification of the lens system is 1/20 and the light collection efficiency to the surface of the subject is 1.0×10^−3^. In this experiment, the CCD was operated in the 8×8 binning mode, and the actual pixel number was 256×256. Taking into account the detection limit, which is determined by dark current and readout noise of the CCD, as well as light collection efficiency, the minimum detectable number of photons on each pixel is estimated ca 100 photon/s/cm^2^ on the surface of the subject under the measurement condition.

### Measurement procedure

All study participants were healthy males in their 20′s without any skin diseases or oral medications. The average time of sleep onset was 23:30 hours (range, 23:00 to 01:00 hours) and that for awakening was 06:15 hours (range, 06:00 to 07:00 hours). No attempt was made to synchronize the sleeping habits of the study participants before the study. From one week before the experiments, subjects were under controlled conditions to maintain regular sleeping hours. Subjects were not allowed to use cosmetics including aftershave lotion. Volunteers had lunch at 12:30 hours and dinner at 18:30 hours. Snacks and cold drinks were allowed between meals, at the study participant's discretion. During body imaging, participants were naked from the waist up. After a slight wipe of the body with lukewarm water, one by one the subjects were invited in the darkroom and where a relax chair was provided. Before photon emission measurements, the subjects were left 15 minutes in the darkroom for dark adaptation. During the dark adaptation, a thermograph to check the surface temperature of the body and a picture of the subject under weak light illumination for focusing are taken. Biophoton emission measurements are taken continuously for 20 minutes by the CCD camera. During measurements, comfortable music was provided for relaxation but sleep was not allowed. At the end of the measurements, thermograph and picture under weak illumination are taken again, together with the oral temperature. Photon emission intensity from the face and upper body was calculated from imaging data. Salivary cortisol levels were measured by radioimmunoassay. For statistical analysis, one-way ANOVAs followed by Bonferroni/Dunn's multiple comparisons were applied. The above experiments are approved by the Ethical Committee of Kobe University Graduate School of Medicine.

## Supporting Information

Figure S1 Photon emission in sleep deprived volunteers. Three volunteers kept in constant light environment (400 lux) without sleep, and photon counts were measured at 25:00 (1:00AM), 28:00 (4:00AM) and 31:00 (7:00AM) (red dots). Note the levels of photon emissions at these time points are much lower than evening value. The values from 10:00–22:00 are adopted from Figure 1H (n = 15, Mean±SD).
(5.65 MB TIF)

Figure S2 Daily change of oral temperature (left; n = 15, Mean±SD) (a), and its correlation to photon emission intensity (b). There was no significant correlation between photon emission and oral temperature (r = 0.1630, p = 0.1682).
(7.76 MB TIF)
