# Supplementary figures and images for: Imaging of Ultraweak Spontaneous Photon Emission from Human Body Displaying Diurnal Rhythm
Source: PLoS One. 2009 Jul 16;4(7):e6256. doi: 10.1371/journal.pone.0006256 (PMC2707605; doi:10.1371/journal.pone.0006256)

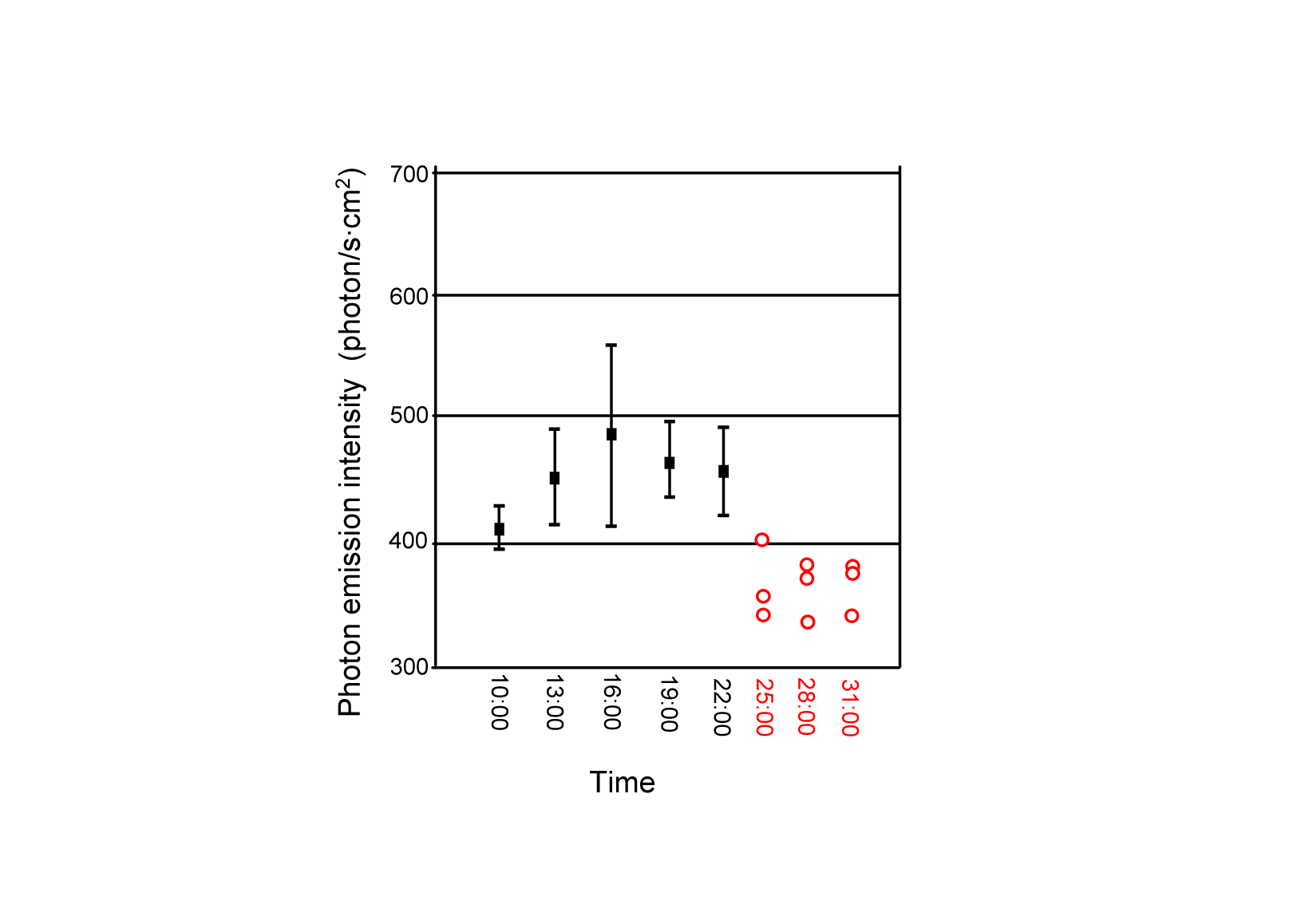

Supplement: Figure S1 — Photon emission in sleep deprived volunteers. Three volunteers kept in constant light environment (400 lux) without sleep, and photon counts were measured at 25:00 (1:00AM), 28:00 (4:00AM) and 31:00 (7:00AM) (red dots). Note the levels of photon emissions at these time points are much lower than evening value. The values from 10:00–22:00 are adopted from Figure 1H (n = 15, Mean±SD). (5.65 MB TIF) [file pone.0006256.s001.tif]

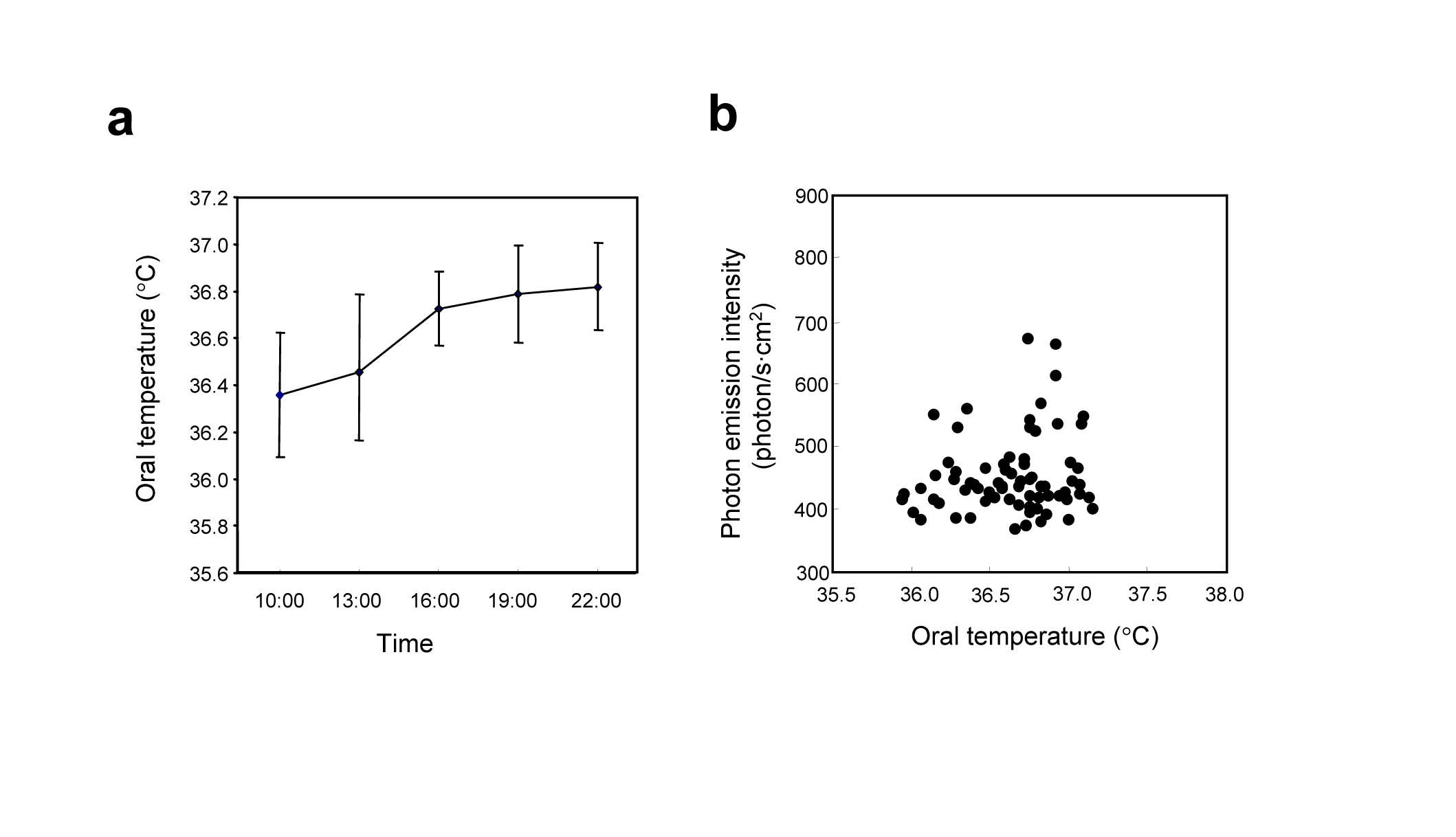

Supplement: Figure S2 — Daily change of oral temperature (left; n = 15, Mean±SD) (a), and its correlation to photon emission intensity (b). There was no significant correlation between photon emission and oral temperature (r = 0.1630, p = 0.1682). (7.76 MB TIF) [file pone.0006256.s002.tif]
